# Spatiotemporal expression pattern of miR-205, miR-26a-5p, miR-17-5p, let-7b-5p, and their target genes during different stages of corpus luteum in Egyptian buffaloes

**DOI:** 10.1186/s43141-022-00320-9

**Published:** 2022-02-25

**Authors:** Sally Ibrahim, Mohamed O. Taqi, A. S. A. Sosa, Al-Shimaa Al-H. H. El-Naby, Karima Gh. M. Mahmoud, Hassan R. H. Darwish, Amal R. Abd El Hameed, M. F. Nawito

**Affiliations:** 1 Department of Animal Reproduction and A.I, Veterinary Research Institute, National Research Centre, Dokki, Giza, 12622 Egypt; 2 Central Laboratory for Agricultural Climate, Agricultural Research Centre, Ministry of Agriculture and Land Reclamation, Dokki, Giza, 12311 Egypt; 3 Department of Theriogenology, Faculty of Veterinary Medicine, Benha University, Banha, Egypt; 4 Cell Biology Department, Institute of Biotechnology Research, National Research Centre, Dokki, 12622 Giza, Egypt

**Keywords:** Buffaloes, Corpus luteum, MicroRNAs, Target genes, Serum steroids

## Abstract

**Background:**

No doubt that the corpus luteum (CL) plays a vital role in the regulation of female cyclicity in mammals. The scenarios among microRNAs (miRNAs) and their target genes and steroid hormones {estradiol (E2) and progesterone (P4)} are required for better understanding the molecular regulation of CL during its formation, maturation, and regression. We aimed to (I) study the changes in the relative abundance of miR-205, miR-26a-5p, miR-17-5p, and *let-7b-5p* and their target genes: LHCGR, CASP3, PCNA, AMH, and PLA2G3, during different stages of corpus luteum in Egyptian buffaloes, and (II) and to address different scenarios between steroid concentrations in the serum and the expression pattern of selected miRNAs and their targets.

**Methods:**

The paired ovaries and blood samples were collected from apparently healthy 50 buffalo cows at a private abattoir. The ovaries bearing CL were macroscopically divided according to their morphological structure and color into hemorrhagic (CLH), developing (CLD), mature (CLM), regressed (CLR), and albicans (CLA). Small pieces from different stages of CL (CLH, CLD, CLM, CLR, and CLA) were cut and immediately kept at − 80 °C for total RNA isolation and qRT-PCR. The serum was separated for steroid level estimation.

**Results:**

The LHCGR was expressed during different stages of CL, and the peak of expression was at the mid-luteal stage. The CASP3 revealed a stage-specific response at different stages of CL. The PCNA has an essential role in cellular proliferation in buffaloes CL. Both expression patterns of PLA2G3 and AMH were found over the various developmental and regression stages. It was noticed that miR-205 is conserved to target LHCGR and CASP3 transcripts. Moreover, CASP3 and AMH were targeted via miR-26a-5p. Additionally, the CASP3 and PLA2G3 were targeted via *let-7b-5p*. The P4 level reached its peak during CLM. There were positive and negative strong correlations between miRNAs (miR-26a-5p and miR-205), target genes (LHCGR and CASP3) during different stages of CL, and steroid hormones in the serum.

**Conclusions:**

Taken together, the orchestrated pattern among miRNAs, target genes, and steroid hormones is essential for maintaining the proper development and function of CL in buffalo cows.

**Supplementary Information:**

The online version contains supplementary material available at 10.1186/s43141-022-00320-9.

## Background

Buffaloes (*Bubalus bubalis*) play a significant role in the agricultural economy of many developing countries, via providing milk, meat, and draught power [1]. The world population of buffaloes was estimated to be ~ 204.3 million according to FAOSTAT [2]. In developing countries, buffaloes are mostly reared under the smallholder farming system [3, 4]. In Egypt, there are around 347.6 thousand buffaloes, which provide 79.15% of milk and 86% of meat production [5]. However, buffaloes are well adaptive to the harsh environment, and their reproductive efficiency is hampered by late puberty, poor oestrous expression, long postpartum anoestrus, long calving interval, poor response to multiple ovulation and embryo transfer (MOET), seasonality, and low conception rates [6–8]. These obstacles are major factors of economic losses to buffalo breeders either in Egypt [3], as well as all over the world [9].

Under normal physiological regulation of ovarian tissues, the development of the mammalian corpus luteum (CL) occurs rapidly in a time-dependent manner within 1 week after ovulation, with morphologic and biochemical changes in the theca interna and granulosa cells of the preovulatory follicle [10]. In the absence of pregnancy, CL should be regressed into non-functional vascular fibrous tissue, in order to animal enters into a new estrous cycle [11]. Improper regulations of luteinization, and angiogenesis during the early luteal phase, result in poor progesterone secretion, which could compromise embryo development, and subsequently reduce female fertility. In case of the absence of pregnancy, inadequate control of the regression process of CL associates with a long female acyclicity [10, 11]. The aberrations of CL function could be a reason for anestrum and delaying animals from entering a new estrous cycle, as well as very early embryonic mortality due to insufficient progesterone secretion [10, 12]. It was reported that CL is an ephemeral gland, which has unique characters, due to its nature of rapid growth, differentiation, cellular remodeling, and death [13, 14]. The complex interactions among growth factors, immune cells, endothelial cells, and cytokines should be highly regulated in order to support the structure and function of CL at each developmental stage (formation, maintenance, and regression) [14–16].

The precise regulation of CL is required during its formation, maturation, and regression to support the structure and function of CL [15]. The regulatory mechanisms of CL function have been well investigated at transcriptional and post-transcriptional levels, in humans [17], mice [18], cattle [19], and ovine species [20]. However, little is known about the transcriptional as well as post-transcription regulations of CL formation, maturation, and regression in Egyptian buffaloes. In a previous study in bovine, it was shown that luteinizing hormone/choriogonadotropin receptor (LHCGR) was expressed in CL throughout the estrous cycle, and the peak of expression was at the mid-luteal stage compared to the other luteal stages [21]. Apoptosis-related cysteine peptidase (CASP3) is one of the genes that are related to programmed cell death (apoptosis) pathways and revealed stage-specific responses during structural luteolysis of CL in bovine [22]. It was demonstrated that the proliferating cell nuclear antigen (PCNA) is a marker for cellular proliferation during the S phase of the cell cycle, in bovine CL [23] and porcine CL [24]. Furthermore, anti-Mullerian hormone (AMH) shows a strong relation to CL numbers after superovulation in high-producing dairy cows [25] and could be used as a biomarker for female fertility as well as superovulation in farm animals [26]. The phospholipase A2 (PLA2G) family plays a role in CL regulation via précis regulation of uterine prostaglandins biosynthesis in bovine [27]. In addition, it was observed that the PLA2G3 transcript was highly expressed in buffalo primary cultured granulosa cells (GC) from different follicular sizes compared to their uncultured one [28]. However, the regulation of CL function could be altered at the post-transcriptional level via microRNAs [29].

MicroRNAs (miRNAs) are small, non-coding RNA molecules that act as post-transcriptional regulators of gene expression by inhibiting translation or degrading mRNA through partial or complete base pairing with the 3′-UTRs of the target mRNAs [30]. The function of miRNAs occurs at multiple hierarchical levels of gene regulatory networks, through targeting hundreds of genes [31]. It has become clear that miRNAs are instrumental players in many events such as inflammatory immune responses, cellular differentiation, apoptosis, and tissue remodeling. The fine-tuning regulation of the expression of these genes is fundamental in directing these processes for normal female fertility [32]. It was stated that multiple arrays of miRNAs are involved in CL development and controlled its function [33]. For instance, the upregulation of miR-34a during the midcycle of luteal cells resulted in the inhibition of cell proliferation and increasing progesterone production [29]. The role of miRNAs could be extended to maintaining the early pregnancy via targeting the genes responsible for CL regression leading to sustaining of progesterone secretion by luteal tissue [34].

Despite the big role of CL in the regulation of female cyclicity, little is known about the regulatory dynamics either at the transcriptional or post-transcription level, which are involved in CL formation, maturation, and regression in buffaloes. Therefore, the current work aimed to (I) investigate the changes in the relative abundance of miR-205, miR-26a-5p, miR-17-5p, and *let-7b-5p* and their target genes: LHCGR, CASP3, PCNA, AMH, and PLA2G3, during different stages of corpus luteum in Egyptian buffaloes, and (II) and to figure out different scenarios between steroid concentrations {estradiol (E2) and progesterone (P4)} in the serum and the expression pattern of selected miRNAs and their target genes. Thus, the current study could give us a better understanding of the molecular basis (transcriptional and/or post-transcription levels) of CL formation, maturation, and regression and subsequently could contribute to improve fertility of Egyptian buffaloes, via maintaining normal physiological function of CL.

## Methods

### Chemicals

All chemicals and reagents were obtained from Qiagen (Hilden, Germany), Thermo Fisher Scientific (Wilmington, USA), unless otherwise stated.

### Samples collection (ovarian tissues and blood) and morphological classification of CL

The ovaries (*n* = 100) were collected in pairs from apparently healthy 50 non-pregnant buffalo cows of unknown reproductive history, at Bahtim private abattoir, Al-Qaliobia, Egypt {from the months of September (2018) to February (2020)}. Directly after slaughtering, blood and ovarian tissues were collected. The ovaries were placed in chilled normal saline (0.9% NaCl) supplemented with 50 μg/l gentamycin (Sigma-Aldrich) and then transported on ice to the laboratory. At the lab, the serum was separated and stored at − 20 °C for the determination of steroid hormones (E2 and P4). Meanwhile, the ovaries were washed 3–4 times in warm saline, disinfected once in 70% ethanol, and then washed again with warm saline. Afterwards, the ovaries bearing CL were macroscopically categorized according to their morphological structure and color into hemorrhagic (CLH) (bloody apex over the rupture point), developing (CLD) (visible vascularization around the periphery of CL), mature (CLM) (fully developed CL with visible vascularization around its periphery; the apex is red or brown, and the rest is grayish), regressed (CLR) (the red or brown color disappeared and no vasculature visible on its surface), and albicans (CLA) (the red or brown color disappeared and the entire CL gray/white) [35–37]. Furthermore, different stages of CL were confirmed via histological examination (data not shown). Small pieces from different stages of CL (CLH, CLD, CLM, CLR, and CLA) were cut and immediately kept at − 80 °C for total RNA isolation (Supplementary Fig. 1).

### In silico analysis for the selected candidate miRNAs

The prediction tools of miRNA such as DIANA-microT v3.0 (http://diana.cslab.ece.ntua.gr/microT/) and miRecords (http://mirecords.biolead.org/) were used for the filtrations of miRNA hits according to their potential relevance for cellular proliferation, apoptosis, regulation of seroidogenesis, and lipid mediators at least in four different search algorithms. We found that miR-205, miR-26a-5p, miR-17-5p, and *let-7b-5p* could be selected as potential targets to investigated genes in the current study.

### RNA isolation and cDNA synthesis

Total RNA was extracted from different stages of CL (six independent biological replicates), using miRNeasy mini kit (Qiagen, Hilden, Germany). We extracted total RNA according to the manufacturer’s protocol. Furthermore, any possible contaminations of genomic DNA were removed via on-column DNA digestion, using the RNase-free DNase kit (Qiagen, Hilden, Germany). The extracted total RNA was subsequently stored at − 80 °C. The concentration of total RNA was determined by Nano-drop 2000/c (Thermo Fisher Scientific, Wilmington, USA), as well as confirmation of the presence of intact RNA, using agarose gel electrophoresis 2%. The samples that had clear 28 and 18S ribosomal RNA bands were selected for quantification. The cDNA was synthesized from the isolated total RNA, using the RevertAid First Strand cDNA Synthesis Kit (Thermo Fisher Scientific, USA). The RNA concentration was adjusted by RNase free water to a maximum volume of 10 μl RNA, then added 1 μl of oligo (dT)_18_ and 1 μl of random primers to have a total volume of 12 μl and kept on ice shortly until prepared a mixture of; 4 μl 5× reaction buffer, 1 μl ribolock RNase inhibitor, 2 μl dNTP mix, and 1 μl revertAid RT. Afterwards, 8 μl of the mixture was added to the RNA samples and then mixed well by pipetting. Thermocycler was adjusted according to the following program: 25 °C for 5 min, 42 °C for 60 min, 70 °C for 5 min, and hold at 4 °C. The synthesized cDNA was confirmed using GAPDH primer in a PCR reaction and then kept at − 20 °C.

### The mRNA and miRNA quantifications

The transcript level of the candidate genes (PCNA, CASP3, LHCGR, PLA2G3, and AMH) was performed in CL obtained at different developmental stages using Maxima SYBR Green/ROX qPCR Master Mix (2×) (Thermo Fisher Scientific, USA), using StratageneMx 3000P instrument (Agilent Technologies, USA). Primer3 as an online tool was used to design the specific primers of the candidate genes (http://bioinfo.ut.ee/primer3-0.4.0/) (Table 1). The master mix which contained cDNA, optimized amount of forward, and reverse primers diluted in water and SYBR green master mix were subjected to thermal cycle initiating with a denaturation step at 95 °C for 10 min, 40 cycles at 95 °C for 15 s, 60 °C for 30 s, and 72 °C for 30 s followed by melting curve analysis in order to verify the amplification specificity. The expression level of housekeeping genes (ACTB and GAPDH) was used for normalization, and their stability over various groups was checked using the NormFinder software [38].

**Table 1:** List of primers used

Gene	Sequence 5′ to 3′	Accession no.	Product size (bp)	Annealing, °C
AMH	F: CTGCTTCACACGAAAGACCC R: GCGTGAGAGTCTCCAGGAAG	XM_006047815.1	170	54
PLA2G3	F: CTGTGTGGAGTGGTACTGGT R: ACTTCTGAGGATGCTGTATCA	XM_006051028.1	176	50
CASP3	F: GACTGTGGTATTGAGACAGACA R: CGTACTTTTTCAGCATCTCAC	XM_006075118.1	175	50
PCNA	F: GTAGTAAAGATGCCTTCTGGTG R: AAGAAGTTCAGGTACCTCAGTG	XM_006047748.1	239	50
LHCGR	F: CATCACCTATGCTATTCAACTG R: TGGTTAAGATGTAGACCTGTGA	NM_174381.1	191	50
GAPDH	F: CTACATGGTCTACATGTTCCAG R: CCTTCTCCATGGTAGTGAAGA	XM_006065800.1	200	50
18S	F: CGCAGCTAGGAATAATGGAA R: TCTGATCGTCTTCGAACCTC	NR_036642	217	55
ACTB	F: GGCATTCACGAAACTACCTT R: CAATCCACACGGAGTACTTG	NM_001290932.1	198	55

For miRNA expression analysis, 10 ng of total RNA was reverse transcribed via MultiScribe reverse transcriptase. The specific primers of candidate miRNAs (*let-7b-5p*, miR-26a-5p, miR-17-5p, and miR205) were separately added for each sample according to the manufacturer’s instructions. The master mix of the real-time PCR was done in a volume of 10 μl, using 0.7 μl of RT product, 0.5 μl of particular primers with probes (Table 2), and TaqMan Universal PCR Master Mix II (Thermo Fisher Scientific, Wilmington, USA). Amplification was carried out using a Stratagene Mx 3000P instrument (Agilent Technologies, USA) with initial denaturation for 10 min at 95 °C, followed by 40 cycles of 15 s at 95 °C and 60 s at 60 °C. The normalization was done using the geometric means of 5S and U6. Finally, the expression level of the mRNAs and miRNAs was expressed as an arbitrary unit calculated by 2^−ΔCt^ = 2^−(Ct target gene-Ct housekeeping gene)^ [39].

**Table 2:** List of miRNAs names, miRbase accession numbers, and their mature sequences

miR name	Accession number	Mature miRNA sequence
*Let7-b-5p*	MIMAT0004331	UGAGGUAGUAGGUUGUGUGGUU
miR-205	MIMAT0003545	UCCUUCAUUCCACCGGAGUCUG
miR-26a-5p	MIMAT0003516	UUCAAGUAAUCCAGGAUAGGCU
miR-17-5p	MIMAT0003815	CAAAGUGCUUACAGUGCAGGUAGU

### Determination of steroid levels (E2 and P4) in the serum

The concentrations of E2 and P4 were determined in the serum using commercial ELISA kits (Chemux BioScience, South San Francisco, USA), according to the manufacturer’s instructions. The optical density (OD) value was measured, using ELISA microplate reader (BioTek ELx800, Vermont, USA), at 450 nm wavelength for both kits. For P4 ELISA kits, sensitivity, intra-assay, and inter-assay coefficients were 0.05 ng/ml, 5.8%, and 9.0%, respectively. For E2 ELISA kits, sensitivity, intra-assay, and inter-assay coefficients were 6.0 pg/ml, 12.1%, and 11.2%, respectively.

### Statistical analysis

The data were represented in means ± SEM and statistically analyzed using the Kruskal-Wallis one-way ANOVA test followed by Dunn’s multiple comparisons test by the GraphPad Prism version 9.0 software (GraphPad Software, Inc., San Diego, CA, USA). The results were considered significant at *P* < 0.05.

## Results

### The expression pattern of candidate genes in a stage-dependent manner

**Fig. 1:**
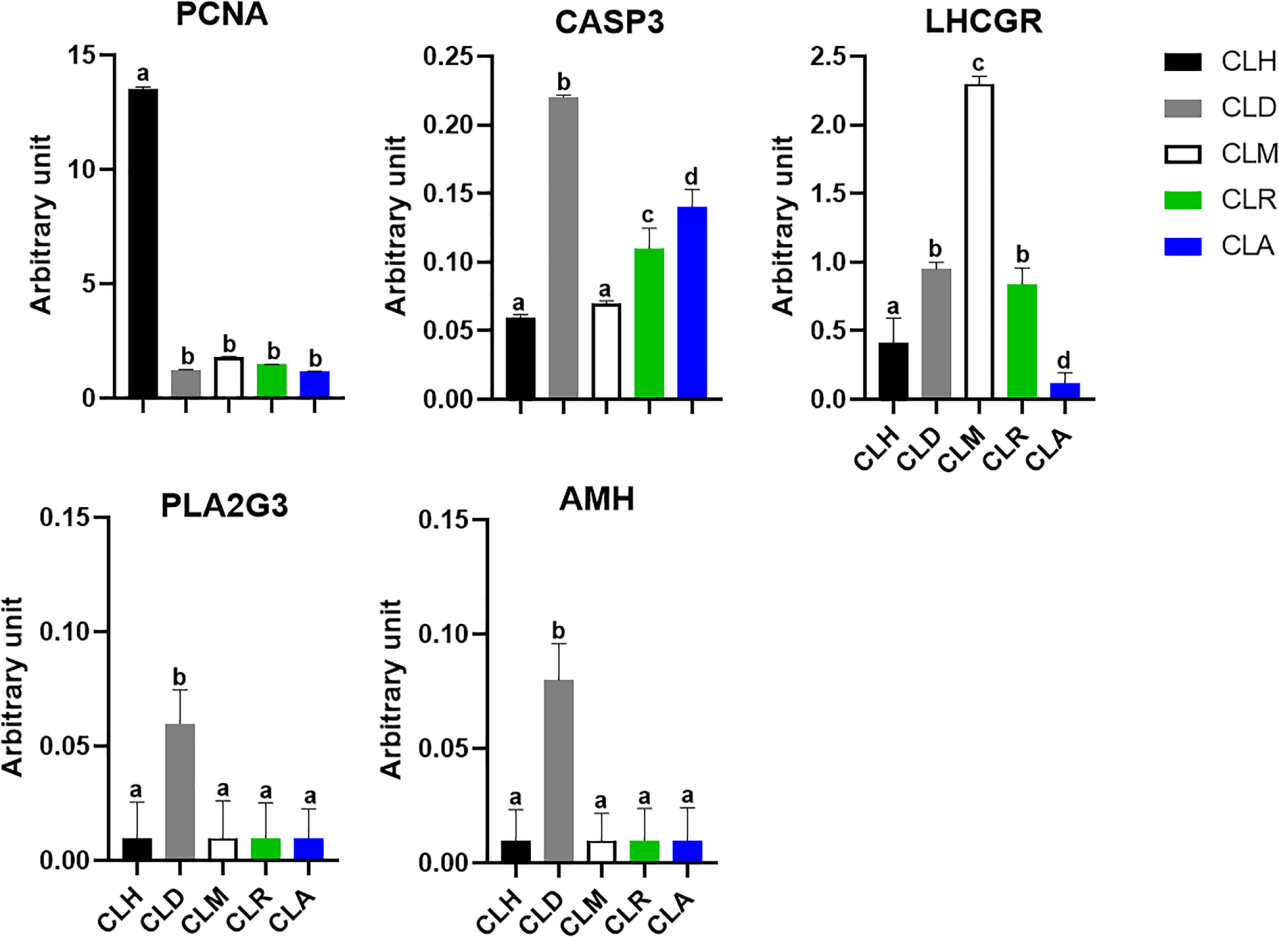
Expression profile of PCNA, CASP3, LHCGR, PLA2G3, and AMH transcripts at different stages of corpus luteum in Egyptian buffaloes. Bars are presented as mean ± SEM. ^a,b,c,d^Statistical differences between different stages of CL: hemorrhagic (CLH), developing (CLD), mature (CLM), regressed (CLR), and albicans (CLA)

The mRNA transcript of the candidate genes (PCNA, CASP3, LHCGR, PLA2G3, AMH) was summarized in Fig. 1. The expression of PCNA was significantly upregulated at the CLH stage compared to other developmental and regression stages. However, the mRNA expression pattern of CASP3 was significantly upregulated at the CLD stage in comparison with the other developmental stages. The same pattern was observed at CLR and CLA, which were significantly different, but less than the CLD stage. Unfortunately, there were no significant differences observed between CLH and CLM. Regarding the LHCGR expression profile, the highest expression level was found at the CLM stage, which was significantly different compared to other stages. On the other hand, the lowest expression pattern of LHCGR was found at CLA compared to the other stages. No significant difference was identified between CLD and CLR stages. Additionally, the same expression pattern of PLA2G3 and AMH was found over the various developmental and regression stages. Their expression was significantly increased at the CLD stage with respect to other developmental stages. Nevertheless, there were no significant differences among CLH, CLM, CLR, and CLA stages in terms of PLA2G3 and AMH mRNA transcriptional levels.

### Spatiotemporal of miRNA expression pattern during different stages of CL in buffalo cows

**Fig. 2:**
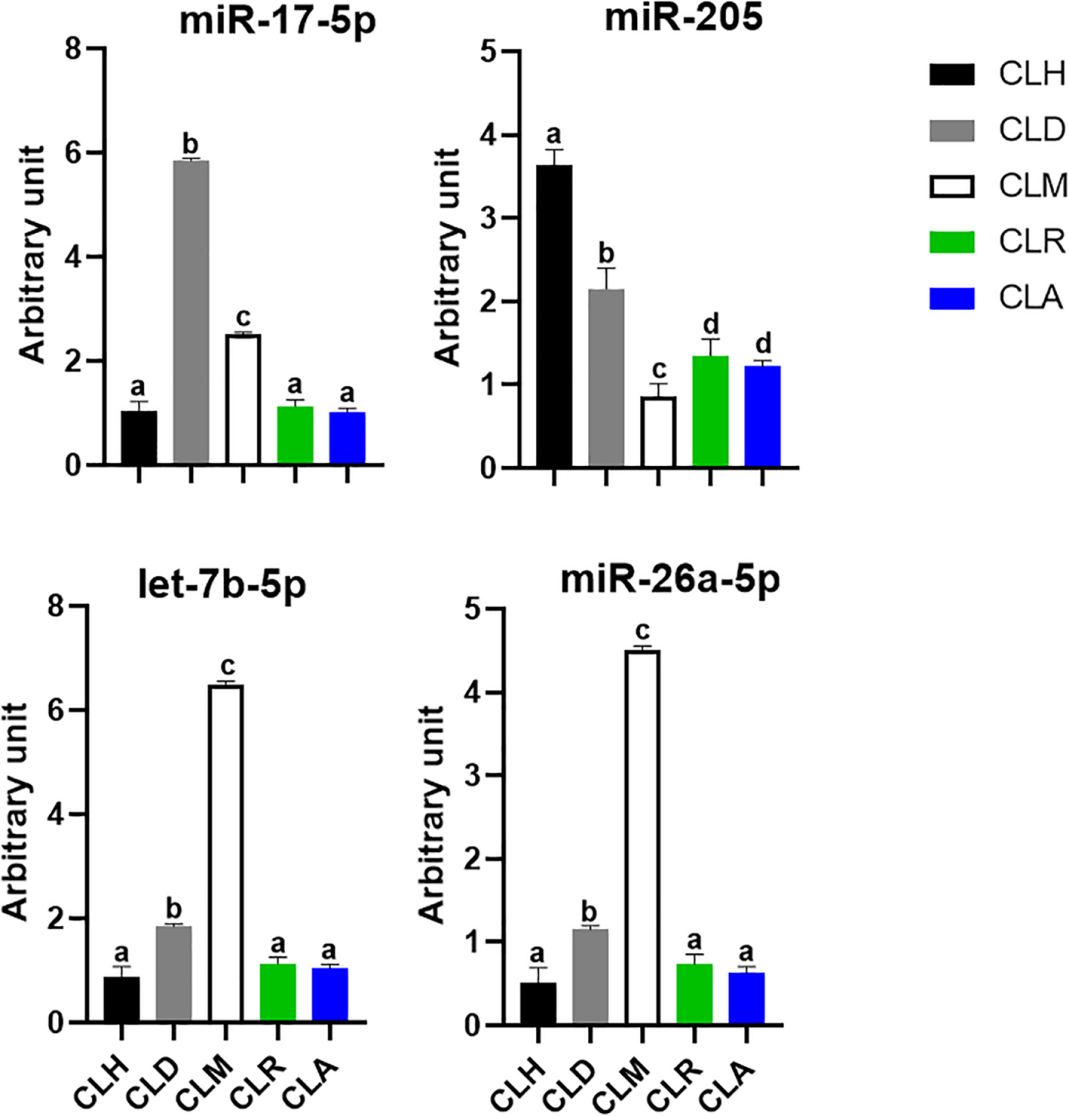
Relative abundance of miR-17-5p, miR-205, *let-7b-5p*, and miR-26a-5p at different stages of corpus luteum in Egyptian buffaloes. Bars are presented as mean ± SEM. ^a,b,c,d^Statistical differences between different stages of CL: hemorrhagic (CLH), developing (CLD), mature (CLM), regressed (CLR), and albicans (CLA)

The in silico analysis of miRNA showed that the PCNA is targeted by miR-17-5p. Furthermore, miR-205 is conserved to target the LHCGR and CASP3 genes. Moreover, CASP3 and AMH are targeted via miR-26a-5p. Additionally, the CASP3 and PLA2G3 were targeted via *let-7b-5p*. Hence, the expression level of those miRNAs was checked, and the results were shown in Fig. 2. Unlike mRNA expression patterns, the expression pattern of miRNAs did not take the same and/or obvious pattern of mRNA over the various developmental stages of corpus luteum. The expression pattern of miR-17-5p was significantly downregulated at the CLH stage compared to CLD and CLM stages. The highest expression level was identified at the CLD stage followed by the CLM stage, which were significantly different from each other. However, the results did not show any significant differences among the CLH, CLR, and CLA stages. Furthermore, the expression of miR-26a-5p and *let-7b-5p* had the same pattern throughout the different developmental stages of corpus luteum in Egyptian buffalo. The expression level of those miRNAs at the CLM stage was increased in comparison with other stages followed by the CLD stage. However, their expression level was significantly decreased at CLH, CLR, and CLA compared to CLM and CLD. Meanwhile, the expression pattern among the three previously mentioned stages was not significant. Regarding the expression pattern of miR-205, the highest expression level was recorded at the CLH stage followed by CLD and subsequently CLR and CLA. The statistical analysis did not reveal any significant differences between CLR and CLA stages. However, the lowest expression level was found at the CLM stage, which was significantly different compared to other developmental stages.

#### Alteration of blood hormonal levels (E2 and P4) in a stage-dependent manner of corpus luteum formation, development, and regression

**Fig. 3:**
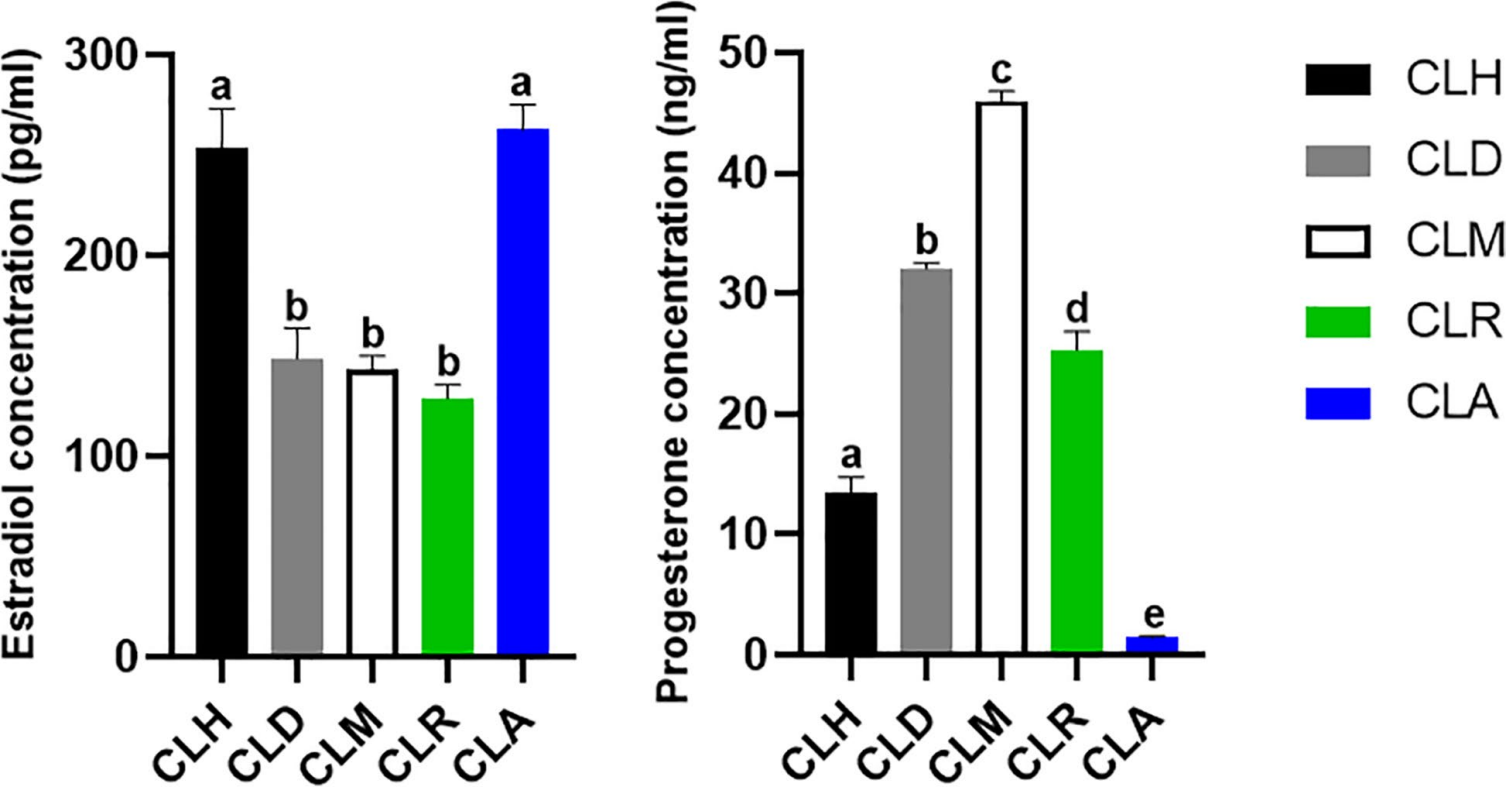
The level of steroid hormones (E2 and P4) in the serum at different stages of corpus luteum in Egyptian buffaloes. Bars are presented as mean ± SEM. ^a,b,c,d^Statistical differences between different stages of CL: hemorrhagic (CLH), developing (CLD), mature (CLM), regressed (CLR), and albicans (CLA)

Following the mRNA and miRNA expression level detection, the concentrations of E2 and P4 in the blood serum were determined, and the results were shown in Fig. 3. The results indicated that the highest concentration of E2 was found at CLH and CLA stages, which were significantly different compared to other stages and insignificant in-between. However, no significant differences were observed among CLD, CLM, and CLR. On the other hand, the concentration of P4 was gradually increased from the CLH stage till the CLM stage. The peak of P4 concentration was recorded at the CLM stage and then gradually decreased till the CLA stage. The lowest level of P4 was found at the CLA stage. Generally, the differences among investigated stages were statistically different in terms of P4 concentration.

### Correlation analysis

At mature CL stage (Table 3), the correlation between the arbitrary units of LHCGR mRNA, miR-205, and serum P4 concentration was positive and strong *r* = 0.9816, *r* = 0.8490, respectively (*P* < 0.01). On the other hand, the correlation between the arbitrary units of LHCGR mRNA, miR-205, and serum E2 concentration did not show any significant differences. At the developing CL stage (Table 3), the correlation between the arbitrary units of miR-26a-5p and serum P4 concentration was negative and strong *r* = − 0.8091 (*P* < 0.01). Furthermore, it was negative and strong correlation *r* = − 0.9650 (*P* < 0.01) between the arbitrary units of CASP3 and serum E2 level (Table 3).

**Table 3:** The correlation between miRNAs, target genes, and serum steroid levels (P4 and E2) during different stages of corpus luteum in buffaloes

Stage	***r***	***P***
***Mature CL***		
**P4**		
LHCGR vs. P4	0.9816	0.01
MiR-205 vs. P4	0.8490	0.01
**E2**		
LHCGR vs. E2	− 0.5284	NS
MiR-205 vs. E2	− 0.1081	NS
***Developing CL***		
**P4**		
miR-26a-5p vs. P4	− 0.8091	0.01
**E2**		
CASP3 vs. E2	− 0.9650	0.01

*r* correlation, *P* probability, *NS* not significant

## Discussion

Currently, it is believed that the corpus luteum formation, function, and regression are controlled by a comprehensive profile of various mRNAs transcripts, which could be targeted and regulated by miRNAs. This regulation is required in order to provide a functional corpus luteum and subsequently maintain the pregnancy in mammals. Most of the previous studies focused on providing a snapshot of the miRNAs’ pattern without investigating their target genes. Hence, we aimed in our study to investigate the alteration of miRNAs (miR-205, miR-26a-5p, miR-17-5p, and *let-7b-5p*) and their target genes (LHCGR, CASP3, PCNA, AMH, and PLA2G3) during different stages of corpus luteum development in Egyptian buffaloes. To our knowledge, this is the first study to investigate the role of mRNA-miRNA interaction in regulating corpus luteum formation, function, and regression in Egyptian buffaloes.

In bovine, several miRNAs were differentially expressed among various developmental stages of CL, which are involved in the vital pathways [33]. Interestingly, the investigated miRNAs: miR-205, miR-26a-5p, miR-17-5p, and *let-7b-5p*, were a part of those miRNAs, which play essential roles in cell proliferation, as well as angiogenesis pathways. The miR-17-5p is found to be involved in cell proliferation, cell function (angiogenesis), and cell regression. In the current study, this miRNA was found to exhibit an opposite pattern with the expression of the PCNA gene, especially during the early and development stages, which is in agreement with the findings of Gecaj et al. [33]. These findings indicated that the low expression of the level of miR-17-5p during the early developmental stage is needed for promoting the proliferation of the corpus luteum. However, mirroring the findings was observed in cancer cells, which showed that miR-17-5p could promote cell proliferation through inhibiting the P21 gene, which is considered a cell cycle inhibitor [40]. On the other hand, the apoptosis process required a functionally CASP3 gene especially during CL regression [41], which is consistent with the current results. Surprisingly, the expression of CASP3 is dramatically increased at the CLD stage and then decreased with gradually increasing during CL regression, due to its functional role during the early stage of leutolysis [42]. Accordingly, the expression of its candidate miRNAs (miR-205, miR-26a-5p, and *let-7b-5p*) showed a reduction during the early stages of CL regression, as has been shown in the current work. Unexpectable, our results showed a downregulation of *let-7b-5p* and miR-26a-5p expression during the CLH, whereas the expression of miR-205 was downregulated during the CLM stage, which were unmatched with the regulation of miRNAs to the CASP3 gene [30]. These results revealed that the regulation of miRNAs to their targets could occur in a stage-dependent manner due to the dynamism of interacting the miRNAs with their targets as well as other factors such as the abundance of miRNAs [43], which needs further investigation.

Several mechanisms are well-coordinated to regulate CL growth and luteolysis. The phospholipase A2 (PLA2) family is a large family that plays a pivotal role in the regulation of an array of cell functions such as lipid metabolism [44], prostaglandins biosynthesis [27], and tumor angiogenesis [45]. As known, the endothelial cells, which represent more than 50% of CL cells count, and blood vessels are essential for CL functions [46], which is supporting our notion. The upregulation of PLA2G3 during CL development is required for its function, as has been shown in the current study. Accordingly, the expression of *let-7b-5p* was downregulated and then upregulated at the CLM stage. Additionally, the prostaglandin F_2_ alpha is involved in the luteolysis process [47]. Therefore, it is required to be synthesized during the late stage of CL development. However, our results were on the opposite side, which showed a downregulation pattern of PLA2G3 and its candidate miRNA during the late stages of CL formation. Furthermore, the same pattern was observed for the AMH gene and its candidate miRNA (miR-26a-5p). The AMH is a member of the transforming growth factor-beta (TGF-β) family. In buffalo, the AMH gene is located in chromosome 9 [26], where it is mapped to chromosome 7 in cattle [48]. It is responsible for controlling the follicle number as well as the selection of dominant follicles. Its expression reached to peak from primordial till secondary follicles and then decline once the selection of dominant follicle [26]. It reduces the sensitivity of granulosa cells to FSH in growing follicles [49]. So far, its physiologicalcal role in CL function remains elusive. Additionally, the inhibation of AMH during CLH and CLA stages could be attributed to high level of E2, which is in agreement with Grynberg et al. [50]. Therefore, our results open a new window for investigating the role of AMH during CL formation and regression, which needs confirmatory research.

To maintain CL function, LHCGR is required due to the binding of luteinizing hormone and human chorionic gonadotropin (hCG) to it. The binding of LH to LHCGR stimulates a signaling cascade leading to produce steroid hormones [51]. Accordingly, our results showed that the peak of LHCGR expression was found in fully functioned CL and then gradually decreased till regression. Meanwhile, the P4 concentration reached its peak during CLM, whereas the concentration was decreased at CLH and CLA. Supporting this notion, the freshly isolated luteinized granulosa cells showed a reduction in LHCGR and then recovered by hCG [52], which resulted in lower P4 production. Moreover, the high levels of LHCGR in the current study were offset by a reduction in miR-205-5p and vice versa, which leads us to lay the cornerstone for understanding the regulatory mechanism of LHCGR and P4 secretion.

Concerning the correlation findings, both positive and negative strong correlations between miRNAs (miR-26a-5p and miR-205), target genes (LHCGR and CASP3) during different stages of CL (developing and mature), and steroid hormones (E2 and P4) in the serum showed the precise regulation to offer a suitable milieu for CL (development and function) [13, 53]

## Conclusions

The dynamics of proliferation (PCNA) and apoptosis (CASP3) genes during CL formation, development, and regression could be regulated via miR-17-5p, miR-205, miR-26a-5p, and *let-7b-5p*, respectively. Furthermore, the same pattern of expression either in mRNA or candidate miRNA was observed for PLA2G3 and AMH, which needs further investigation. Additionally, the LHCGR, which showed differential alteration in its expression over different stages of CL development, is regulated via miR-205 that contributed to the alteration in P4 concentration. Ultimately, harmony in mRNA-miRNA regulations is essential for functional corpus luteum, which could contribute to the understanding of the molecular mechanism behind CL development. This study opens a new window for understanding the molecular mechanism of CL development in order to map the gene network and their epigenetics regulatory mechanisms (Supplementary Fig. 2).

## Supplementary Information

**Additional file 1: Figure S1**. Schematic diagram shown the whole experimental design. CLH: hemorrhagic corpus luteum, CLD: developing corpus luteum, CL M: mature corpus luteum, and CLA: corpus luteum albicans.

**Additional file 2: Figure S2**. Schematic model summarized the scenario among miRNAs, mRNAs and serum P4 level at developing corpus luteum (CLD) as well as mature corpus luteum (CLM) stages in Egyptian buffalo cows.

## Data Availability

The data that support the findings of this study are available from the corresponding author upon reasonable request.

## Abbreviations

CL: Corpus luteum
miRNAs: MicroRNAs
E2: Estradiol
P4: Progesterone
CLH: Hemorrhagic CL
CLD: Developing CL
CLM: Mature CL
CLR: Regressed CLR
CLA: Albicans CL
MOET: Multiple ovulation and embryo transfer
LHCGR: Luteinizing hormone/choriogonadotropin receptor
CASP3: Apoptosis-related cysteine peptidase
PCNA: Proliferating cell nuclear antigen
AMH: Anti-Mullerian hormone
PLA2G3: Phospholipase A2 G3
