# Supplementary figures and images for: Spatiotemporal expression pattern of miR-205, miR-26a-5p, miR-17-5p, let-7b-5p, and their target genes during different stages of corpus luteum in Egyptian buffaloes
Source: J Genet Eng Biotechnol. 2022 Feb 25;20:37. doi: 10.1186/s43141-022-00320-9 (PMC8881532; doi:10.1186/s43141-022-00320-9)

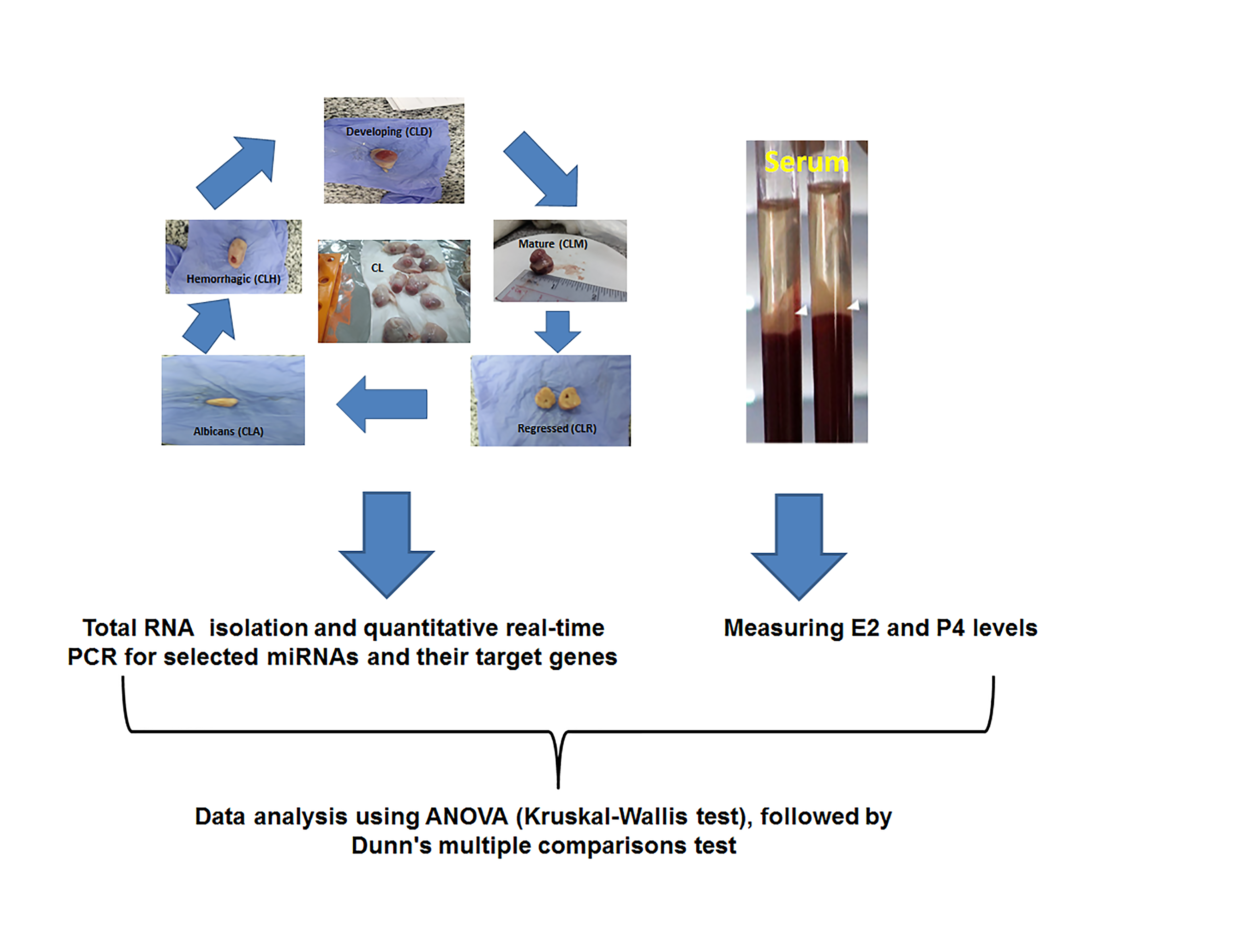

Supplement: Supplementary file 1 — Additional file 1: Figure S1. Schematic diagram shown the whole experimental design. CLH: hemorrhagic corpus luteum, CLD: developing corpus luteum, CL M: mature corpus luteum, and CLA: corpus luteum albicans. [file 43141_2022_320_MOESM1_ESM.tif]

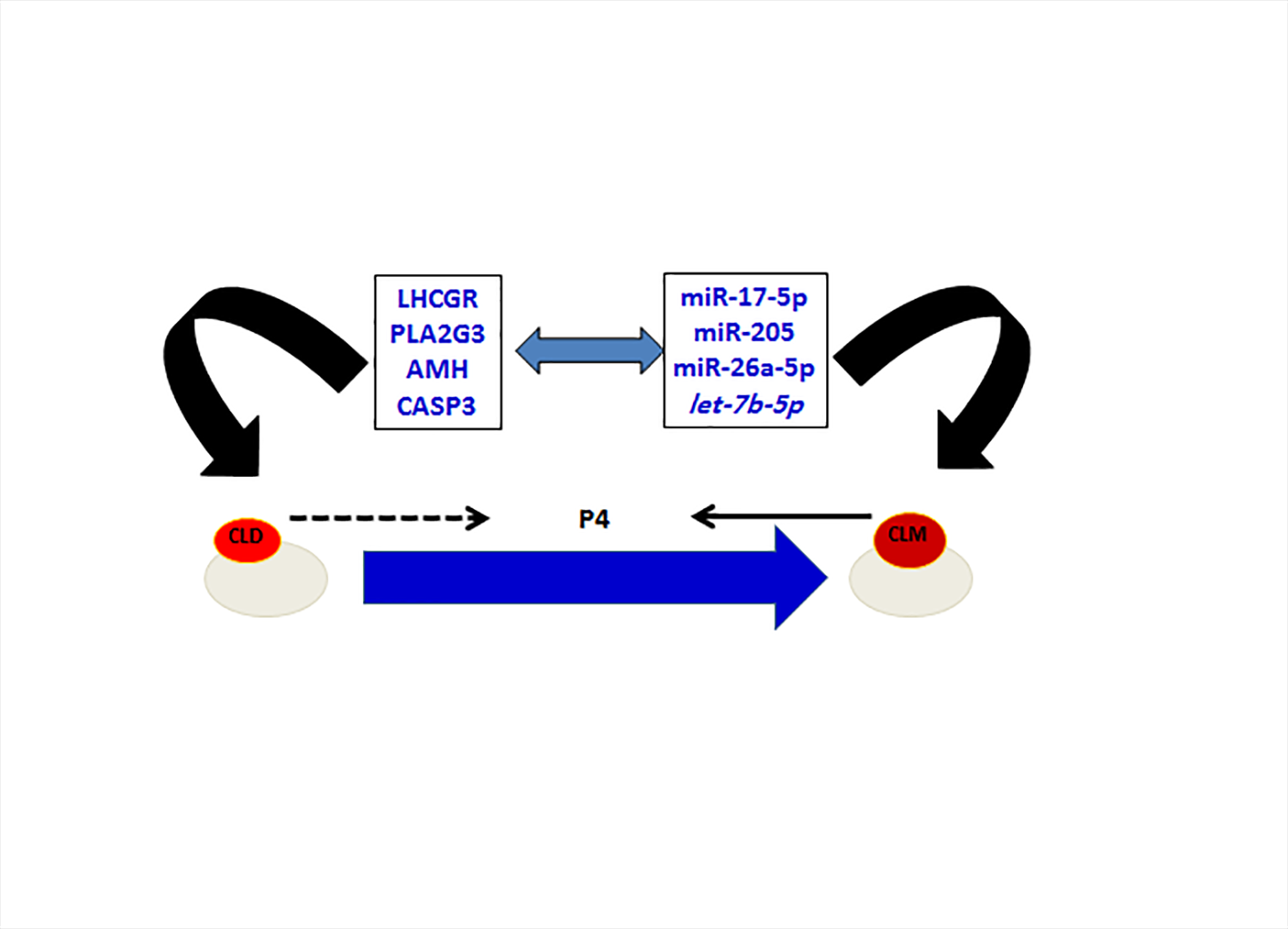

Supplement: Supplementary file 2 — Additional file 2: Figure S2. Schematic model summarized the scenario among miRNAs, mRNAs and serum P4 level at developing corpus luteum (CLD) as well as mature corpus luteum (CLM) stages in Egyptian buffalo cows. [file 43141_2022_320_MOESM2_ESM.tif]
